# 

**Published:** 1853-10-01

**Authors:** 


### WORKS RECEIVED.

ΨΥΧΗ. *A Discourse on the Birth and Pilgrimage of Thought.* By W. C. Dendy, Esq. London: Longman, Brown, Green, & Longmans. 1853. —This elegant and charmingly written work will be analysed at length in the January number of our Journal.

*Notes and Narratives of a Six Years' Mission, principally among the Dens of London.* By R. W. Vanderkiste. London: James Nisbett, 1853.—This work will be reviewed in our next number, with Mr. Hill's valuable work on Crime.

*Elements of Psychological Medicine.* By Daniel Noble, F.R.C.S. London: J. Churchill, 1853.—In our next.

*Popular Errors on the Subject of Insanity Examined and Exposed.* By J. F. Duncan, A.M., M.D. Dublin: James McGlashan; and John Churchill, London, 1853.—In our next.

*A Manual of Artistic Anatomy.* By R. Knox, M.D. London: H. Renshaw, 1852.—In our next.

*Ospizio di S. Benedetto, in Pesaro, Statistica sul Movimento degli Alienati, dall' anno della sua Fondazione,* 1829, *a tutto Giugno dell' anno corrente,* 1852. Del Dottore Giuseppe Girolami, Medico Direttore.—In our next.

*The Limit of Demonstrative Science Considered.* By H. L. Mansell, D.D. Oxford: 1853.—In our next.

*The Prevalent Treatment of Disease.* By F. C. Skey, F.R.S. London: 1853.—An exceedingly well written pamphlet, fraught with valuable instruction.

*Tenth Annual Report of the State Lunatic Asylum of New York.* Albany: 1853.—In our next.

*The Philosophy of Vital Motion.* By C. B. Radcliffe, M.D. London: J. Churchill.—We are reluctantly obliged to postpone the insertion of our review of Dr. Radcliffe's valuable work until a future number.

*Prolegomena Logica. An Enquiry into the Psychological Character of Logical Processes.* By H. L. Mansell, D.D. Oxford: 1851.—This valuable work will be reviewed in our next.

*Études Cliniques. Traité Théorique et Pratique des Maladies Mentales, &c.* Par M. Morel, Médecin-en-Chef de l'Asile d'Aliénés de Mareville, &c. Tomes 1 and 2. Paris: 1853.—In our next.

*Observations on the Remittent* (so called) *Yellow Fever of the West Indies.* By David L. Finlay, L.R.C.S.I. & L.A. Dublin: Fannin & Co., 1853. —An interesting and well written pamphlet.

---

We have received several Reports of County Lunatic Asylums, too late to be noticed in our present number.

The American Journal of Insanity will be noticed at length in our January number. It contains many valuable articles.

We have received several Reports of Irish Asylums, which must stand over for the present.

---

### MEDICAL SOCIETY OF LONDON.

The first meeting of the winter session of this Society takes place on the 8th of October, when Dr. Forbes Winslow, the president, will read a paper "On some Unrecognised Forms of Mental Disorder."

### INDEX TO VOL. VI.

ABOLITION of private asylums, 615
Act, the new, for the regulation of the care and management of lunatics, 590
Action, automatic and volitional, difference between, 386
Aged, the, their soporous condition considered, 230
——— repuerescence of, denied, 233
Alternation, love of, one of the radical passions, 158
American asylums for the insane, 605
Amusements and employment of patients, 15, 20, 24, 36, 39, 581
Anatomical physicians, 572
Asylums, effects of, on noisy patients, 2
——— large, expense of, 406

Belfast Lunatic Asylum, and the chaplaincy question, 280
Belgium, position of the insane in, 257
Belief, as distinguished from experience, 371
Berkeley, bishop, his philosophical writings, 209
Bethlem Hospital, absence of records of insanity in, 98, 109
——— annual report on, 29, 595
——— few but apparently curable cases admitted into, 87
——— has contributed nothing to medical science, 98
——— how governed, 87
——— in 1851 compared with 1815, 97
——— neglect of the medical staff, 90
——— report of the Commissioners in Lunacy on, 129
——— state of criminal lunatics in, 41
——— to be under supervision of Commissioners in Lunacy, 594
——— want of an infirmary in, 91, 109
Biographical writings, their effects on the public mind, 200
Boismont, A. Brierre de, on hallucinations, 161
Boismont, A. Brierre de, on the development of insanity as influenced by civilization, 242
Boyd, Dr., his remarks on the medical treatment of insanity, 21
Brain, the extent of the surface of the, 264
——— the human surface of the, in proportion to its bulk, less than that of inferior animals, 265
British asylums for the insane, 1, 543
Brown, Dr. Thomas, his distribution of the mind, 216

Chancery lunatics, return of the House of Commons as to, 450
Charlesworth, death of Dr. E. P., 314
Children, epidemic mental diseases of, 118
——— their proneness to belief rather than disbelief considered, 368
Civilization, its influence on the development of insanity, 242
Clairvoyance considered, 170, 377
Classification of patients, 20
Climate, effects of, on the mind, 165, 173
Colney-Hatch Asylum, cost of, 405
Commentary on medical and moral life, by W. Cooke, M.D., 113
Commissioners in Lunacy and Visiting Justices, conflicting authority of, 475
——— Seventh annual report of the, 459
——— their inquiry into the state of Bethlem Hospital, 83, 129
Confinement, separate, at Pentonville Prison, results of, by John B. Burt, assistant chaplain, 179
Consulting physicians to county asylums, importance of, 472
Cooke, W., M.D., on medical and moral life, 113
Cottage System, objections to the, 479
County Asylums, architecture of, 404
——— disadvantages of, 3
——— responsible head of should be the medical officer, 408, 411
——— Commissioners' suggestions for the staff of, 465

County Asylums, salaries of superior officers of, 468
Court of Aldermen, hereditary Governors of Bethlem, 87
Crime and Punishment, by F. Hill, 284
Crime, suggestions for the prevention of, 284
——— social position of women in relation to, 286
Criminal class, often found among people of weak intellects, 185
Criminal lunatics at Dundrum, 541
——— number of, 482
——— periodical inquiries suggested as to their mental state, 83
——— present state of the law as to, 41
——— separate treatment of, 481
Cumming, Mrs. Catherine, death of, 458

Davy, Dr., on the proximate causes of insanity, 563
Definition in mental science, vagueness of, 163
Dendy, W. C. Esq. on the remedial influence of the mind, 268
Denham, Rev. J. F., on the metaphysics of education, 210
Descartes, his services to science, 571
Dickson, his remarks on the treatment in the Manchester Asylum, 9
Diseases, bodily and mental, their connection, 350
District lunatic asylums in Ireland, names of principal officers of, 534
Duality of human dynamism, 228, 574
Duncan, James F., M.D., on moral insanity, unaccompanied by any obvious intellectual aberration, 274.

Eccentricity, distinguished from madness, 421
Education, the effects of, on the senses, 398
——— metaphysics of, 210
Elements of psychology, 317
Employment, the good effects of, on the insane, 15, 20, 24, 36, 39, 581.
Epidemic mental diseases of children, 118
Epilepsy, in the insane, treatment of, 553
——— peculiar cases of, 21

Fear, effects of, on the mind, 269, 270
Feuchtersleben, Ernest Von, M.D., on the dietetics of the soul, 342
Fiction, prose and poetic, present state of, 201
Fontanelle, insenescence of, in old age, 227
Fourier, Charles, on the passions of the soul, 147

Galt, John M., M.D., on the recreation and amusements of the insane, 581
Genius seldom combined with common sense, 501
German writers, their peculiarities, 353
Glasgow Royal Asylum, report on the, 34
Guislain, J., on insanity, 256, 418

Habit, as to sensation, thought, and motion, 382, 397-399
——— psychologically considered, 380
Hallucinations, by A. Brierre de Boismont, 161
——— comparative frequency of, by night and day, 177
——— etiology of, 171
——— frequent before epilepsy, 169
——— physical causes of, 172
——— studied in reference to psychology, history, and religion, 174
——— treatment of, 177
Hanwell Asylum, eighth report of the visitors of, 437
Haydon, a psychological study, 501
——— progress of his insanity, 521
Healing process, more slow in a defeated army than in the conquerors, 271
Hegel, his theory of the soul, 344
Hereditary insanity, 23
Hill, Frederick, on crime and punishment, 284
Home education, effects of, 489
Hüberts, Dr. F. R., on mental diseases in Denmark, 441
Human dynamism, 228, 574
Hume and Spurzheim, value of their works, 344

Ideas, association of, 339
Illusions, how effected by surrounding events, 171.
Impotent, in some sense all people are, 150
Imprisonment, its effects on the mind, 182
India, insanity in, 356
Inductive evidence, rationale of, 367
Insane, advantages of occupation to the, 366
——— British Institutions for the, 1,543
——— persons, new contrivance for administering food to, 311
——— recreations and amusements of the, 581
Insanity, advantages of an acquaintance with, 257
——— how averted, 494
——— in India, 356
——— influence of the seasons on, 359
——— importance of the early detection of, 456

Insanity, its causes, 35-39, 492-563
——— lectures on, by Guislain, Professor in the University of Ghent, 256, 418
Insenescence in old age, instances of, 227-231
Inspection of asylums necessary to check abuses, 100
Intellectual and moral character of the age, 191
Intellectual and zoonomic existence, relative progress of, 221-239
Intellectual development, relation between the extent of the surface of the brain, and the degree of, 265-267
Intellectual principle, the, does not decline from the apogee of the vital force, 224, 225
——— exact point of the decline of, during old age, impossible to be assigned, 227
——— how affected by mental disease, 222
Intellectual powers, analysis of the, 331
Intuition and perception, psychological identity between, 337
Ireland, lunatic asylums in, 125
——— Sixth General Report of Lunatic Asylums in, 528

Johnston, Capt., the case of, 40
——— his statement, 43

Keepers in Bethlem Hospital, their conduct, 91

Law of lunacy, contemplated alterations in the, 304
Legal cases in lunacy, 289
Literature, present state of, in England, 195
Logic for the Million, or the Art of Reasoning, by a Fellow of the Royal Society, 209
Lordat, M., Professor of Physiology in the University of Montpellier, on Mental Dynamics, 221, 566
Lunacy, contemplated alterations in the law of, 304
——— incurable, suggestions with reference to cases of, 315
——— in England in 1847 and 1852, 463
——— state of, 459
——— in Ireland, extent of, 533
——— legal cases in, 289
Lunatic asylums, amount of medical supervision of, 411
——— in Ireland, 125, 528
——— pauper, 403
——— position of the matron with respect to the medical officer of, 415
Lunatic asylums, visiting physician of, desirable in all cases, 415
Lunatics, causes of their death, 7
——— cost of maintenance of, 406
——— found by inquisition, number of, 462
——— statistics of, 492
——— the New Act for the regulation of the care and treatment of, 590
——— treatment of, by seclusion, 17

M'Cormac, Henry, M.D., on moral sanitary economy, 482
Medicine, mental dynamics in relation to the science of, 221, 566
Medical men, absence of religious conviction among, denied, 117
Medical officers of asylums and hospitals for the insane, the association of, 453
——— qualifications for, 409
——— authority of, 411-417
——— small remuneration of, 405-414
Medical staff of Bethlem Hospital, duties of, 88-109
Melancholy often induced by protracted illusion, 168
——— the phases and causes of, 435
Mental affections, classification of, 425
Mental diseases in Denmark, statistics of, 441
Mental dynamics in relation to the science of medicine, 221, 566
Mental faculties, classification of the, 327
Mental philosophy, introduction to, by G. Ramsay, 209
——— popularity of the study of, 209
Mental process, rapidity of, 217
Mental statistics of Pentonville prison, 187
Mental vigour in cases of premature death, 223
Metaphysics, the popular study of, 209
Mind and body, their reciprocal action, 350, 355
Mind and emotions, 313
Mind, effects of imprisonment on the, 182
——— functions of the, present acquaintance with, 204
——— genesis of the, and its connexion with the body, 328
——— ambiguity of modern writers on the, 347
——— pathological influence of the, 268, 272
——— philosophy of the, by Dr. T. Brown, 217
——— workings of the, in a reverie, 165
Moral cause of mental disorders greater among women than men, 245
Moral insanity, case of, unaccompanied by any obvious symptoms of intellectual aberration, 274

Moral insanity, instances of, 250
Moral treatment, effects of, on the insane, 3-9
Monomania and hallucination, combination of, 168
Morel, J. D., on the elements of psychology, 317

Occult sciences, 567

Papillionic memories, 158
Pasquier, M. Etienne, intellectual power of during old age, 231
Passions, analysis of the, 151
Pathology of insanity, 599
Pauper Lunatic Asylums, 403
Pauper lunatics, separate treatment of, 464
Pentonville Prison, insanity in, compared with other prisons, 183
——— present system compared with that formerly in use, 185-190
Perception and intuition, psychological identity between, 336
Philosophy, mental and natural, their relationship, 320
Philosophical literature, character of, 203
Physical science, distinguished from philosophy, 570
Prison discipline, 179, 288
Proximate causes of insanity, the, 563
Psychology, classification of the powers of the mind necessary to the study of, 218
——— distinguished from physical sciences, 570
Psychological science, present and past state of, compared, 206
——— writers on, 208
Psychologists, beneficial effects of their researches, 206
Psychotherapeia, or the remedial influence of the mind, 268
Puerperal mania, case of, in India, 364

Ragged children, education of, 488
Ramsay, George, B.M., on mental philosophy, 209
Reason, fundamental laws of, pervade the mental and material world, 322
Recreation and amusement of the insane, 581
Reflective energy, 169
Religion, effects of, on the passions, 114
Religious conviction, want of, among medical men denied, 117
Religious instruction of the insane, 15-29
Resident medical officer of Bethlem Hospital, duties of, 90, 102
Restraint in cases of insanity, remarks on, 9, 11, 12, 16
Reverie, the workings of the mind in, 165

Scotland, lunatic asylums in, 32
Schelling, his views of the universe, 320
Science, ill effects of an imperfect knowledge of, 197
Second childhood, a popular delusion, 232
Senescence of bodily and mental power considered, 238
Senile imbecility, 230
Sensation, nature of, 334
——— process of, 397
Simonds, John A., M.D., on habit, 380
Social life, two different states of, 153
Society, the present compared with the past state of, 484
Somnambulism defined, 170
Soul, dietetics of the, 342
—— how it differs from the mind, 346
—— how regarded by the ancient writers, 343
—— the passions of the, 147
Spurzheim and Hume, value of their works, 344
Statistical tables of insanity, value of, 31
St. Luke's Hospital, 28, 478
Suggestions with reference to cases of incurable lunacy, 315
Suicidal insanity, 23
Suicide, Haydon's, remarks on, 521
——— more frequent in urban than rural districts, 247
——— when most prevalent, 351
Superintendents of asylums, non-medical, 351

Table-turning and spirit-rapping, 601
Taylor, Tom, his Life of Haydon, 501
Thought, laws of, as distinguished from analogical inductions, 375
Treasurer of Bethlem Hospital, powers of, 88

Universe, four ascending stages of the, 323

Valerianate of zinc, use of, in cases of epilepsy, 8
Visiting justices of asylums, exercise of their authority, 477

Warren, Samuel, F.R.S., on the intellectual and moral character of the present age, 191
Williams, Watkin, on the philosophy of evidence, 367
Wise, Thomas N., M.D., on insanity in Bengal, 356
Women, status of, 486
——— the intellectual capacity of, 211

Zoonomic and intellectual existence, relative progress of, 221, 239

